# Aqua­(9,10-dioxoanthracene-1,5-disul­fonato-κ*O* ^1^)bis­(1,10-phenanthroline-κ^2^ *N*,*N*′)nickel(II)

**DOI:** 10.1107/S1600536810034252

**Published:** 2010-09-04

**Authors:** Ying-Yu Cao

**Affiliations:** a Key Laboratory for Green Chemical Technology of the Ministry of Education, School of Chemical Engineering and Technology, Tianjin University, Tianjin 300072, People’s Republic of China and College of Chemistry and Life Science, Tianjin Normal University, Tianjin 300387, People’s Republic of China

## Abstract

In the mononuclear title complex, [Ni(C_14_H_6_O_8_S_2_)(C_12_H_8_N_2_)_2_(H_2_O)], the Ni^II^ atom is in a distorted octa­hedral coordination formed by four N atoms from two chelating 1,10-phenanthroline ligands and by two O atoms, one from a 9,10-dioxoanthracene-1,5-disulfonate ligand and the other from a water mol­ecule. An intra­molecular O—H⋯O hydrogen bond occurs. In the crystal, inter­molecular O—H⋯O hydrogen bonds link the mononuclear complexes into chains extending parallel to [010]. Furthermore, π–π stacking inter­actions [centroid–centroid distance = 3.5696 (6) Å] stabilize the crystal structuure.

## Related literature

For the structures and applications of organo­sulfonate-based metal complexes, see: Dai *et al.* (2006 ▶); Cui *et al.* (2007 ▶); Zhao *et al.* (2007 ▶); Jia *et al.* (2009 ▶); Chen *et al.* (2002 ▶); Yang *et al.* (2007 ▶). For mol­ecular self-assembly by non-covalent inter­actions in crystal engineering, see: Hunter (1993 ▶). 
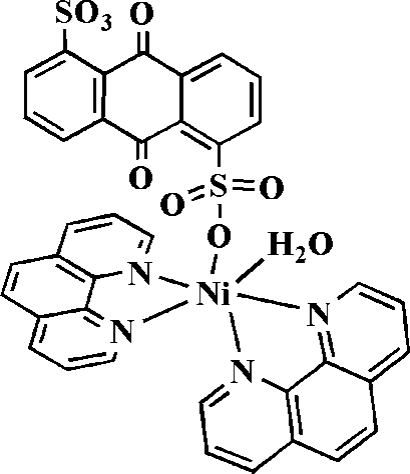


## Experimental

### 

#### Crystal data

[Ni(C_14_H_6_O_8_S_2_)(C_12_H_8_N_2_)_2_(H_2_O)]
*M* *_r_* = 803.44
Triclinic, 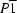

*a* = 8.8784 (19) Å
*b* = 12.802 (3) Å
*c* = 14.723 (3) Å
α = 98.016 (3)°
β = 100.520 (3)°
γ = 91.380 (2)°
*V* = 1627.1 (6) Å^3^
*Z* = 2
Mo *K*α radiation
μ = 0.79 mm^−1^
*T* = 294 K
0.34 × 0.32 × 0.28 mm

#### Data collection

Bruker APEXII CCD area-detector diffractometer
Absorption correction: multi-scan (*SADABS*; Sheldrick, 1996 ▶) *T*_min_ = 0.774, *T*_max_ = 0.808
8942 measured reflections
5693 independent reflections
4243 reflections with *I* > 2σ(*I*)
*R*_int_ = 0.020

#### Refinement

*R*[*F* ^2^ > 2σ(*F* ^2^)] = 0.038
*wR*(*F* ^2^) = 0.104
*S* = 1.05
5693 reflections
487 parameters
H-atom parameters constrained
Δρ_max_ = 0.43 e Å^−3^
Δρ_min_ = −0.43 e Å^−3^

### 

Data collection: *APEX2* (Bruker, 2003 ▶); cell refinement: *SAINT* (Bruker, 2001 ▶); data reduction: *SAINT*; program(s) used to solve structure: *SHELXS97* (Sheldrick, 2008 ▶); program(s) used to refine structure: *SHELXL97* (Sheldrick, 2008 ▶); molecular graphics: *SHELXTL* (Sheldrick, 2008 ▶) and *DIAMOND* (Brandenburg & Berndt, 1999 ▶); software used to prepare material for publication: *SHELXL97*.

## Supplementary Material

Crystal structure: contains datablocks I, global. DOI: 10.1107/S1600536810034252/bt5331sup1.cif

Structure factors: contains datablocks I. DOI: 10.1107/S1600536810034252/bt5331Isup2.hkl

Additional supplementary materials:  crystallographic information; 3D view; checkCIF report

## Figures and Tables

**Table 1:** Hydrogen-bond geometry (Å, °)

*D*—H⋯*A*	*D*—H	H⋯*A*	*D*⋯*A*	*D*—H⋯*A*
O9—H9*A*⋯O3	0.85	1.90	2.704 (3)	156
O9—H9*B*⋯O5^i^	0.85	1.97	2.823 (3)	179

Symmetry code: (i) 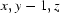.

**Fig. 1.**
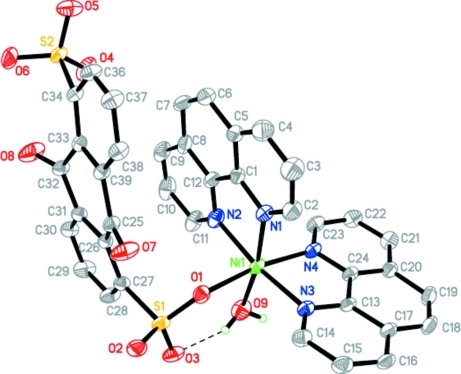
The molecular structure of the title compound with intramolecular hydrogen-bonding interactions. Displacement ellipsoids are drawn at the 30% probability level. Only H atoms involved in hydrogen bonds have been included.

**Fig. 2.**
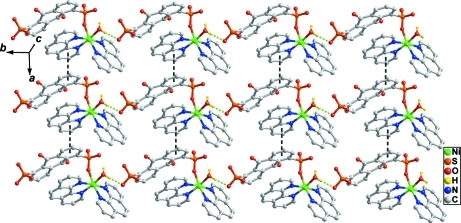
two-dimensional supramolecular nework of I formed by hydrogen-bonding and π–π stacking interactions.

## supplementary crystallographic information

### Comment

Organosulfonate-based metal complexes with popular nitrogen-containing coligands have recently drawn more attention due to their adjustable coordination ability and interesting applications in optical and catalysis (Cui *et al.*, 2007; Yang *et al.*, 2007). In this regard, arenedisulfonates have rigid spacers and potentially multiple binding sites, showing various coordination modes ranging from terminally monodentate to bridging hexadentate (Chen *et al.*, 2002). Simultaneously, their six oxygen atoms in sulfonate groups can also form H-bonds, which readily result in high-dimensional supramolecular architectures (Hunter, 1993). As part of our investigations on the coordination chemistry of organosulfonate ligands (Dai *et al.* 2006; Jia *et al.*, 2009; Zhao *et al.*, 2007), herein, we report the crystal structure of a Ni^II^ complex with 1,10-phenanthroline and 9,10-dioxo-anthracene-1,5-disulfonate ligands (I).

The mononuclear structure of I was shown in Fig. 1. The unique Ni ^II^atom in I is six-coordinated by four N atoms from two chelating 1,10-phenanthroline ligands, one monodentate sulfonate O atom from one 9,10-dioxoanthraquinone-1,5-disulfonate anion, and one coordinated water molecule, exhibiting a slightly distorted octahedral coordination geometry (Table 1). Acting as a typically chelating ligand, two 1,10-phenanthroline ligands coordinate to the central Ni^II^ atom in an asymmetric modes. In contrast, 9,10-dioxoanthraquinone-1,5-disulfonate binds the Ni^II^ ion in a monodentate mode. Additionally, one intramolecular hydrogen bond between the coordinated water molecule and sulfonate group of 9,10-dioxoanthraquinone-1,5-disulfonate was observed (Table 2), which obviously helps to stabilize the mononuclear entity.

In the packing structure of I, one interligand O—H ···O hydrogen bond between the aqua and 9,10-dioxoanthraquinone-1,5-disulfonate link the discrete monoclear structures into a one-dimensional chain (Fig. 2 and Table 2). Furthermore, the neighboring chains are stacked into a two-dimensional layer by π··· π stacking interactions between the pyridine ring of 1,10-phenanthroline and the benzene ring of 9,10-dioxoanthraquinone-1,5-disulfonate. The centroid-centroid distance and the dihedral angle between the two rings are 3.5696 (6) Å and 3.969 (1)°, respectively.

### Experimental

The title complex was synthesized by heating a mixture of NiAc_2_.4H_2_O (49.77 mg,0.2 mmol), 1,10-phenanthroline (39.64 mg,0.2 mmol), Anthraquinone-1,5-disulfonic acid disodium salt (82.4 mg, 0.2 mmol) and H_2_O (10 ml) in a 23 ml Teflon-lined autoclave under autogenous pressure at 140¯C for two days. Yellow block-shaped crystals suitable for X-ray analysis were obtained in 40% yield. Anal. Calcd. for: C_38_H_24_N_4_NiO_9_S_2_: C, 56.81, H, 3.01, N, 6.97%. Found: C, 56.87; H, 3.01; N, 7.01%.

### Refinement

H atoms were located in difference maps, but were subsequently placed in calculated positions and treated as riding, with C – H = 0.93 and O – H = 0.85 Å. All H atoms were allocated displacement parameters related to those of their parent atoms [*U*_iso_(H) = 1.2 *U*_eq_(C) or 1.5 *U*_eq_(O)].

### Crystal data

**Table:**

[Ni(C_14_H_6_O_8_S_2_)(C_12_H_8_N_2_)_2_(H_2_O)]	*Z* = 2
*M**_r_* = 803.44	*F*(000) = 824
Triclinic, *P*1	*D*_x_ = 1.640 Mg m^−^^3^
Hall symbol: -P 1	Mo *K*α radiation, λ = 0.71073 Å
*a* = 8.8784 (19) Å	Cell parameters from 2813 reflections
*b* = 12.802 (3) Å	θ = 2.3–25.2°
*c* = 14.723 (3) Å	µ = 0.79 mm^−^^1^
α = 98.016 (3)°	*T* = 294 K
β = 100.520 (3)°	Block, yellow
γ = 91.380 (2)°	0.34 × 0.32 × 0.28 mm
*V* = 1627.1 (6) Å^3^	

### Data collection

**Table:**

Bruker APEXII CCD area-detector diffractometer	5693 independent reflections
Radiation source: fine-focus sealed tube	4243 reflections with *I* > 2σ(*I*)
graphite	*R*_int_ = 0.020
phi and ω scans	θ_max_ = 25.0°, θ_min_ = 2.0°
Absorption correction: multi-scan (*SADABS*; Sheldrick, 1996)	*h* = −10→10
*T*_min_ = 0.774, *T*_max_ = 0.808	*k* = −15→12
8942 measured reflections	*l* = −17→16

### Refinement

**Table:**

Refinement on *F*^2^	Primary atom site location: structure-invariant direct methods
Least-squares matrix: full	Secondary atom site location: difference Fourier map
*R*[*F*^2^ > 2σ(*F*^2^)] = 0.038	Hydrogen site location: inferred from neighbouring sites
*wR*(*F*^2^) = 0.104	H-atom parameters constrained
*S* = 1.05	*w* = 1/[σ^2^(*F*_o_^2^) + (0.0542*P*)^2^ + 0.3249*P*] where *P* = (*F*_o_^2^ + 2*F*_c_^2^)/3
5693 reflections	(Δ/σ)_max_ = 0.001
487 parameters	Δρ_max_ = 0.43 e Å^−^^3^
0 restraints	Δρ_min_ = −0.43 e Å^−^^3^

### Special details

**Table:**

Geometry. All e.s.d.'s (except the e.s.d. in the dihedral angle between two l.s. planes) are estimated using the full covariance matrix. The cell e.s.d.'s are taken into account individually in the estimation of e.s.d.'s in distances, angles and torsion angles; correlations between e.s.d.'s in cell parameters are only used when they are defined by crystal symmetry. An approximate (isotropic) treatment of cell e.s.d.'s is used for estimating e.s.d.'s involving l.s. planes.
Refinement. Refinement of *F*^2^ against ALL reflections. The weighted *R*-factor *wR* and goodness of fit *S* are based on *F*^2^, conventional *R*-factors *R* are based on *F*, with *F* set to zero for negative *F*^2^. The threshold expression of *F*^2^ > σ(*F*^2^) is used only for calculating *R*-factors(gt) *etc*. and is not relevant to the choice of reflections for refinement. *R*-factors based on *F*^2^ are statistically about twice as large as those based on *F*, and *R*- factors based on ALL data will be even larger.

### Fractional atomic coordinates and isotropic or equivalent isotropic displacement parameters (Å^2^)

**Table:**

	*x*	*y*	*z*	*U*_iso_*/*U*_eq_	
Ni1	0.51255 (4)	0.67299 (3)	0.76204 (2)	0.03729 (13)	
S1	0.20477 (9)	0.73215 (6)	0.85480 (5)	0.0398 (2)	
O1	0.3705 (2)	0.74179 (15)	0.85534 (13)	0.0405 (5)	
O2	0.1640 (2)	0.76635 (16)	0.94350 (14)	0.0476 (5)	
O3	0.1423 (2)	0.62602 (16)	0.81432 (15)	0.0525 (6)	
O4	0.3113 (3)	1.19722 (17)	0.55562 (14)	0.0599 (7)	
O5	0.4395 (3)	1.36031 (18)	0.63825 (16)	0.0649 (7)	
O6	0.1714 (3)	1.33736 (18)	0.62472 (18)	0.0662 (7)	
O7	0.3099 (3)	0.95751 (17)	0.92421 (14)	0.0589 (7)	
O8	0.0471 (3)	1.1338 (2)	0.6416 (2)	0.0840 (10)	
O9	0.3412 (3)	0.56089 (17)	0.70180 (16)	0.0596 (6)	
H9A	0.2799	0.5630	0.7401	0.089*	
H9B	0.3718	0.5007	0.6830	0.089*	
N1	0.6648 (3)	0.80251 (18)	0.81146 (17)	0.0409 (6)	
N2	0.4355 (3)	0.7717 (2)	0.66457 (17)	0.0459 (6)	
N3	0.6219 (3)	0.58737 (18)	0.86155 (16)	0.0373 (6)	
N4	0.6551 (3)	0.58717 (18)	0.68469 (16)	0.0390 (6)	
C1	0.6355 (3)	0.8838 (2)	0.7615 (2)	0.0401 (7)	
C2	0.7799 (4)	0.8171 (3)	0.8840 (2)	0.0510 (8)	
H2	0.8028	0.7618	0.9182	0.061*	
C3	0.8673 (4)	0.9109 (3)	0.9112 (3)	0.0669 (11)	
H3	0.9470	0.9176	0.9627	0.080*	
C4	0.8372 (4)	0.9926 (3)	0.8631 (3)	0.0687 (11)	
H4	0.8940	1.0563	0.8822	0.082*	
C5	0.7204 (4)	0.9808 (2)	0.7847 (3)	0.0516 (9)	
C6	0.6834 (4)	1.0610 (3)	0.7278 (3)	0.0618 (10)	
H6	0.7405	1.1250	0.7418	0.074*	
C7	0.5677 (5)	1.0457 (3)	0.6545 (3)	0.0583 (10)	
H7	0.5454	1.1000	0.6190	0.070*	
C8	0.4773 (4)	0.9488 (3)	0.6291 (2)	0.0501 (9)	
C9	0.3536 (4)	0.9285 (3)	0.5550 (2)	0.0595 (10)	
H9	0.3231	0.9811	0.5190	0.071*	
C10	0.2769 (5)	0.8328 (3)	0.5347 (2)	0.0639 (10)	
H10	0.1962	0.8188	0.4840	0.077*	
C11	0.3212 (4)	0.7549 (3)	0.5915 (2)	0.0551 (9)	
H11	0.2684	0.6893	0.5773	0.066*	
C12	0.5131 (4)	0.8670 (2)	0.6827 (2)	0.0422 (7)	
C13	0.7236 (3)	0.5218 (2)	0.8298 (2)	0.0358 (7)	
C14	0.6111 (4)	0.5946 (2)	0.9512 (2)	0.0462 (8)	
H14	0.5416	0.6399	0.9738	0.055*	
C15	0.7008 (4)	0.5364 (3)	1.0124 (2)	0.0516 (9)	
H15	0.6929	0.5451	1.0751	0.062*	
C16	0.7991 (4)	0.4675 (2)	0.9804 (2)	0.0451 (8)	
H16	0.8568	0.4273	1.0206	0.054*	
C17	0.8134 (3)	0.4573 (2)	0.8862 (2)	0.0408 (7)	
C18	0.9110 (4)	0.3856 (2)	0.8449 (2)	0.0495 (8)	
H18	0.9665	0.3404	0.8810	0.059*	
C19	0.9248 (4)	0.3818 (3)	0.7543 (2)	0.0510 (8)	
H19	0.9893	0.3343	0.7293	0.061*	
C20	0.8404 (3)	0.4508 (2)	0.6967 (2)	0.0418 (7)	
C21	0.8543 (4)	0.4540 (3)	0.6035 (2)	0.0506 (8)	
H21	0.9205	0.4104	0.5758	0.061*	
C22	0.7691 (4)	0.5223 (3)	0.5541 (2)	0.0515 (8)	
H22	0.7775	0.5258	0.4926	0.062*	
C23	0.6699 (4)	0.5864 (2)	0.5963 (2)	0.0459 (8)	
H23	0.6110	0.6310	0.5611	0.055*	
C24	0.7396 (3)	0.5191 (2)	0.73459 (19)	0.0367 (7)	
C25	0.2713 (3)	0.9862 (2)	0.84869 (19)	0.0361 (7)	
C26	0.1617 (3)	0.9217 (2)	0.77210 (18)	0.0316 (6)	
C27	0.1177 (3)	0.8148 (2)	0.77205 (19)	0.0360 (7)	
C28	0.0029 (3)	0.7658 (3)	0.7013 (2)	0.0501 (9)	
H28	−0.0268	0.6956	0.7012	0.060*	
C29	−0.0685 (4)	0.8182 (3)	0.6313 (2)	0.0580 (10)	
H29	−0.1466	0.7838	0.5852	0.070*	
C30	−0.0244 (3)	0.9214 (2)	0.6293 (2)	0.0448 (8)	
H30	−0.0714	0.9564	0.5813	0.054*	
C31	0.0898 (3)	0.9730 (2)	0.69854 (19)	0.0365 (7)	
C32	0.1328 (3)	1.0862 (2)	0.6930 (2)	0.0409 (7)	
C33	0.2762 (3)	1.1352 (2)	0.75234 (18)	0.0326 (6)	
C34	0.3511 (3)	1.2275 (2)	0.73629 (19)	0.0348 (6)	
S2	0.31237 (9)	1.28400 (6)	0.62861 (5)	0.0406 (2)	
C36	0.4761 (4)	1.2716 (2)	0.8016 (2)	0.0437 (8)	
H36	0.5253	1.3328	0.7917	0.052*	
C37	0.5305 (4)	1.2280 (2)	0.8810 (2)	0.0462 (8)	
H37	0.6133	1.2607	0.9245	0.055*	
C38	0.4619 (3)	1.1357 (2)	0.8958 (2)	0.0409 (7)	
H38	0.4998	1.1050	0.9484	0.049*	
C39	0.3358 (3)	1.0890 (2)	0.83153 (18)	0.0338 (6)	

### Atomic displacement parameters (Å^2^)

**Table:**

	*U*^11^	*U*^22^	*U*^33^	*U*^12^	*U*^13^	*U*^23^
Ni1	0.0464 (2)	0.0297 (2)	0.0329 (2)	0.00424 (16)	−0.00302 (17)	0.00803 (16)
S1	0.0423 (4)	0.0375 (4)	0.0388 (4)	−0.0023 (3)	−0.0022 (3)	0.0168 (3)
O1	0.0382 (12)	0.0387 (11)	0.0413 (12)	0.0021 (9)	−0.0029 (9)	0.0083 (9)
O2	0.0522 (13)	0.0541 (14)	0.0403 (12)	0.0037 (10)	0.0078 (10)	0.0211 (10)
O3	0.0591 (14)	0.0380 (12)	0.0569 (14)	−0.0133 (10)	−0.0046 (11)	0.0179 (10)
O4	0.0929 (18)	0.0480 (14)	0.0317 (12)	0.0215 (12)	−0.0056 (11)	0.0007 (10)
O5	0.0696 (16)	0.0626 (16)	0.0610 (15)	−0.0220 (13)	−0.0017 (13)	0.0266 (13)
O6	0.0672 (16)	0.0620 (16)	0.0826 (18)	0.0345 (13)	0.0226 (13)	0.0398 (14)
O7	0.0852 (17)	0.0502 (14)	0.0338 (12)	−0.0166 (12)	−0.0153 (11)	0.0189 (10)
O8	0.0617 (16)	0.0684 (17)	0.112 (2)	−0.0115 (13)	−0.0398 (15)	0.0560 (16)
O9	0.0673 (16)	0.0399 (13)	0.0631 (15)	−0.0023 (11)	−0.0020 (12)	−0.0025 (11)
N1	0.0456 (15)	0.0353 (14)	0.0424 (15)	0.0039 (11)	0.0067 (12)	0.0098 (11)
N2	0.0568 (17)	0.0450 (16)	0.0339 (14)	0.0126 (13)	0.0004 (12)	0.0078 (12)
N3	0.0424 (14)	0.0345 (14)	0.0342 (14)	0.0002 (11)	0.0022 (11)	0.0094 (11)
N4	0.0491 (15)	0.0313 (13)	0.0349 (14)	0.0028 (11)	−0.0014 (11)	0.0107 (11)
C1	0.0440 (18)	0.0347 (17)	0.0459 (18)	0.0096 (13)	0.0160 (14)	0.0098 (14)
C2	0.050 (2)	0.0425 (19)	0.056 (2)	−0.0013 (15)	−0.0033 (16)	0.0076 (16)
C3	0.063 (2)	0.053 (2)	0.074 (3)	−0.0076 (18)	−0.009 (2)	0.001 (2)
C4	0.057 (2)	0.043 (2)	0.101 (3)	−0.0075 (17)	0.008 (2)	0.003 (2)
C5	0.050 (2)	0.0363 (18)	0.076 (2)	0.0096 (15)	0.0291 (18)	0.0134 (17)
C6	0.065 (2)	0.040 (2)	0.091 (3)	0.0085 (17)	0.035 (2)	0.020 (2)
C7	0.080 (3)	0.039 (2)	0.075 (3)	0.0194 (18)	0.048 (2)	0.0287 (18)
C8	0.064 (2)	0.054 (2)	0.0438 (19)	0.0303 (17)	0.0265 (17)	0.0215 (16)
C9	0.082 (3)	0.062 (2)	0.046 (2)	0.033 (2)	0.0245 (19)	0.0241 (18)
C10	0.073 (3)	0.082 (3)	0.0352 (19)	0.032 (2)	−0.0012 (17)	0.0129 (19)
C11	0.072 (2)	0.052 (2)	0.0368 (18)	0.0181 (17)	−0.0031 (17)	0.0065 (15)
C12	0.0542 (19)	0.0375 (17)	0.0407 (18)	0.0121 (14)	0.0183 (15)	0.0119 (14)
C13	0.0379 (16)	0.0292 (15)	0.0385 (17)	−0.0043 (12)	−0.0022 (13)	0.0119 (13)
C14	0.052 (2)	0.051 (2)	0.0358 (18)	0.0010 (15)	0.0057 (14)	0.0111 (15)
C15	0.055 (2)	0.064 (2)	0.0380 (18)	−0.0036 (17)	0.0037 (15)	0.0222 (16)
C16	0.0443 (18)	0.0462 (19)	0.0455 (19)	−0.0021 (15)	−0.0034 (15)	0.0257 (15)
C17	0.0410 (17)	0.0363 (17)	0.0443 (18)	−0.0039 (13)	−0.0013 (14)	0.0167 (14)
C18	0.0481 (19)	0.0383 (18)	0.061 (2)	0.0087 (15)	−0.0043 (16)	0.0209 (16)
C19	0.0480 (19)	0.0457 (19)	0.060 (2)	0.0142 (15)	0.0047 (16)	0.0141 (17)
C20	0.0421 (17)	0.0362 (17)	0.0460 (19)	0.0029 (13)	0.0036 (14)	0.0081 (14)
C21	0.057 (2)	0.050 (2)	0.0433 (19)	0.0054 (16)	0.0079 (16)	0.0026 (16)
C22	0.067 (2)	0.053 (2)	0.0340 (17)	0.0019 (17)	0.0070 (16)	0.0054 (15)
C23	0.060 (2)	0.0423 (18)	0.0330 (17)	0.0031 (15)	−0.0010 (15)	0.0106 (14)
C24	0.0413 (17)	0.0303 (15)	0.0359 (16)	−0.0041 (13)	−0.0017 (13)	0.0083 (13)
C25	0.0376 (16)	0.0366 (16)	0.0329 (16)	0.0030 (12)	−0.0010 (12)	0.0110 (13)
C26	0.0288 (14)	0.0365 (16)	0.0300 (15)	0.0019 (12)	0.0023 (12)	0.0103 (12)
C27	0.0328 (15)	0.0403 (17)	0.0346 (16)	−0.0023 (12)	0.0000 (12)	0.0135 (13)
C28	0.0462 (19)	0.0463 (19)	0.052 (2)	−0.0137 (15)	−0.0117 (15)	0.0193 (16)
C29	0.0443 (19)	0.067 (2)	0.053 (2)	−0.0188 (16)	−0.0200 (16)	0.0197 (18)
C30	0.0352 (17)	0.055 (2)	0.0425 (18)	−0.0031 (14)	−0.0078 (14)	0.0216 (15)
C31	0.0312 (15)	0.0426 (17)	0.0361 (16)	0.0005 (12)	0.0013 (12)	0.0142 (13)
C32	0.0374 (16)	0.0435 (18)	0.0420 (17)	0.0010 (13)	−0.0030 (14)	0.0207 (14)
C33	0.0372 (16)	0.0306 (15)	0.0297 (15)	0.0062 (12)	0.0031 (12)	0.0061 (12)
C34	0.0412 (17)	0.0287 (15)	0.0331 (16)	0.0018 (12)	0.0030 (13)	0.0051 (12)
S2	0.0494 (5)	0.0337 (4)	0.0392 (4)	0.0050 (3)	0.0030 (3)	0.0133 (3)
C36	0.056 (2)	0.0315 (16)	0.0402 (17)	−0.0077 (14)	0.0010 (15)	0.0055 (13)
C37	0.058 (2)	0.0389 (18)	0.0350 (17)	−0.0071 (15)	−0.0050 (15)	0.0004 (14)
C38	0.0498 (18)	0.0385 (17)	0.0309 (16)	0.0007 (14)	−0.0022 (13)	0.0062 (13)
C39	0.0400 (16)	0.0330 (15)	0.0281 (15)	0.0039 (12)	0.0038 (12)	0.0068 (12)

### Geometric parameters (Å, °)

**Table:**

Ni1—O9	2.051 (2)	C11—H11	0.9300
Ni1—N3	2.066 (2)	C13—C17	1.410 (4)
Ni1—N1	2.074 (2)	C13—C24	1.431 (4)
Ni1—N2	2.077 (2)	C14—C15	1.399 (4)
Ni1—N4	2.086 (3)	C14—H14	0.9300
Ni1—O1	2.145 (2)	C15—C16	1.354 (5)
S1—O2	1.431 (2)	C15—H15	0.9300
S1—O3	1.458 (2)	C16—C17	1.404 (4)
S1—O1	1.472 (2)	C16—H16	0.9300
S1—C27	1.804 (3)	C17—C18	1.427 (5)
O4—S2	1.432 (2)	C18—C19	1.355 (4)
O5—S2	1.449 (2)	C18—H18	0.9300
O6—S2	1.436 (2)	C19—C20	1.439 (4)
O7—C25	1.212 (3)	C19—H19	0.9300
O8—C32	1.209 (3)	C20—C24	1.400 (4)
O9—H9A	0.8501	C20—C21	1.406 (4)
O9—H9B	0.8501	C21—C22	1.369 (4)
N1—C2	1.326 (4)	C21—H21	0.9300
N1—C1	1.359 (3)	C22—C23	1.388 (5)
N2—C11	1.326 (4)	C22—H22	0.9300
N2—C12	1.356 (4)	C23—H23	0.9300
N3—C14	1.331 (4)	C25—C26	1.484 (4)
N3—C13	1.349 (4)	C25—C39	1.494 (4)
N4—C23	1.330 (4)	C26—C31	1.406 (3)
N4—C24	1.367 (3)	C26—C27	1.414 (4)
C1—C5	1.404 (4)	C27—C28	1.384 (4)
C1—C12	1.425 (4)	C28—C29	1.375 (4)
C2—C3	1.383 (5)	C28—H28	0.9300
C2—H2	0.9300	C29—C30	1.374 (4)
C3—C4	1.348 (5)	C29—H29	0.9300
C3—H3	0.9300	C30—C31	1.381 (4)
C4—C5	1.392 (5)	C30—H30	0.9300
C4—H4	0.9300	C31—C32	1.506 (4)
C5—C6	1.421 (5)	C32—C33	1.477 (4)
C6—C7	1.335 (5)	C33—C39	1.404 (3)
C6—H6	0.9300	C33—C34	1.411 (4)
C7—C8	1.428 (5)	C34—C36	1.380 (4)
C7—H7	0.9300	C34—S2	1.813 (3)
C8—C9	1.390 (5)	C36—C37	1.379 (4)
C8—C12	1.405 (4)	C36—H36	0.9300
C9—C10	1.355 (5)	C37—C38	1.377 (4)
C9—H9	0.9300	C37—H37	0.9300
C10—C11	1.407 (4)	C38—C39	1.389 (4)
C10—H10	0.9300	C38—H38	0.9300
			
O9—Ni1—N3	96.61 (9)	C16—C15—C14	120.0 (3)
O9—Ni1—N1	170.98 (9)	C16—C15—H15	120.0
N3—Ni1—N1	92.15 (9)	C14—C15—H15	120.0
O9—Ni1—N2	91.57 (10)	C15—C16—C17	119.5 (3)
N3—Ni1—N2	171.08 (10)	C15—C16—H16	120.2
N1—Ni1—N2	79.83 (10)	C17—C16—H16	120.2
O9—Ni1—N4	87.47 (10)	C16—C17—C13	117.0 (3)
N3—Ni1—N4	80.16 (9)	C16—C17—C18	124.2 (3)
N1—Ni1—N4	96.19 (10)	C13—C17—C18	118.9 (3)
N2—Ni1—N4	96.71 (9)	C19—C18—C17	121.7 (3)
O9—Ni1—O1	90.17 (9)	C19—C18—H18	119.2
N3—Ni1—O1	91.38 (9)	C17—C18—H18	119.2
N1—Ni1—O1	87.44 (9)	C18—C19—C20	120.4 (3)
N2—Ni1—O1	92.15 (9)	C18—C19—H19	119.8
N4—Ni1—O1	170.88 (8)	C20—C19—H19	119.8
O2—S1—O3	112.60 (13)	C24—C20—C21	117.7 (3)
O2—S1—O1	113.95 (12)	C24—C20—C19	119.0 (3)
O3—S1—O1	110.91 (13)	C21—C20—C19	123.3 (3)
O2—S1—C27	109.11 (13)	C22—C21—C20	119.1 (3)
O3—S1—C27	104.16 (13)	C22—C21—H21	120.5
O1—S1—C27	105.37 (12)	C20—C21—H21	120.5
S1—O1—Ni1	133.04 (12)	C21—C22—C23	119.7 (3)
Ni1—O9—H9A	105.1	C21—C22—H22	120.1
Ni1—O9—H9B	114.9	C23—C22—H22	120.1
H9A—O9—H9B	117.0	N4—C23—C22	123.1 (3)
C2—N1—C1	117.4 (3)	N4—C23—H23	118.5
C2—N1—Ni1	129.7 (2)	C22—C23—H23	118.5
C1—N1—Ni1	112.88 (19)	N4—C24—C20	122.7 (3)
C11—N2—C12	117.8 (3)	N4—C24—C13	116.9 (3)
C11—N2—Ni1	129.2 (2)	C20—C24—C13	120.4 (3)
C12—N2—Ni1	112.95 (19)	O7—C25—C26	121.8 (2)
C14—N3—C13	118.3 (3)	O7—C25—C39	119.5 (2)
C14—N3—Ni1	128.4 (2)	C26—C25—C39	118.7 (2)
C13—N3—Ni1	113.18 (18)	C31—C26—C27	118.5 (2)
C23—N4—C24	117.6 (3)	C31—C26—C25	117.3 (2)
C23—N4—Ni1	130.2 (2)	C27—C26—C25	124.1 (2)
C24—N4—Ni1	112.18 (19)	C28—C27—C26	118.9 (2)
N1—C1—C5	122.7 (3)	C28—C27—S1	115.7 (2)
N1—C1—C12	117.1 (3)	C26—C27—S1	125.4 (2)
C5—C1—C12	120.2 (3)	C29—C28—C27	121.7 (3)
N1—C2—C3	122.9 (3)	C29—C28—H28	119.1
N1—C2—H2	118.5	C27—C28—H28	119.1
C3—C2—H2	118.5	C30—C29—C28	120.0 (3)
C4—C3—C2	120.0 (3)	C30—C29—H29	120.0
C4—C3—H3	120.0	C28—C29—H29	120.0
C2—C3—H3	120.0	C29—C30—C31	120.0 (3)
C3—C4—C5	119.6 (3)	C29—C30—H30	120.0
C3—C4—H4	120.2	C31—C30—H30	120.0
C5—C4—H4	120.2	C30—C31—C26	120.9 (3)
C4—C5—C1	117.3 (3)	C30—C31—C32	117.6 (2)
C4—C5—C6	123.7 (3)	C26—C31—C32	121.5 (2)
C1—C5—C6	118.9 (3)	O8—C32—C33	122.7 (3)
C7—C6—C5	120.9 (3)	O8—C32—C31	118.8 (3)
C7—C6—H6	119.5	C33—C32—C31	118.5 (2)
C5—C6—H6	119.5	C39—C33—C34	119.0 (2)
C6—C7—C8	122.0 (3)	C39—C33—C32	117.1 (2)
C6—C7—H7	119.0	C34—C33—C32	123.8 (2)
C8—C7—H7	119.0	C36—C34—C33	118.5 (2)
C9—C8—C12	116.6 (3)	C36—C34—S2	116.2 (2)
C9—C8—C7	125.0 (3)	C33—C34—S2	124.8 (2)
C12—C8—C7	118.5 (3)	O4—S2—O6	115.06 (15)
C10—C9—C8	120.7 (3)	O4—S2—O5	113.50 (16)
C10—C9—H9	119.7	O6—S2—O5	109.79 (15)
C8—C9—H9	119.7	O4—S2—C34	105.34 (13)
C9—C10—C11	119.1 (3)	O6—S2—C34	108.66 (14)
C9—C10—H10	120.5	O5—S2—C34	103.64 (13)
C11—C10—H10	120.5	C37—C36—C34	122.2 (3)
N2—C11—C10	122.3 (3)	C37—C36—H36	118.9
N2—C11—H11	118.8	C34—C36—H36	118.9
C10—C11—H11	118.8	C38—C37—C36	119.8 (3)
N2—C12—C8	123.4 (3)	C38—C37—H37	120.1
N2—C12—C1	117.1 (2)	C36—C37—H37	120.1
C8—C12—C1	119.5 (3)	C37—C38—C39	119.6 (3)
N3—C13—C17	122.9 (3)	C37—C38—H38	120.2
N3—C13—C24	117.5 (2)	C39—C38—H38	120.2
C17—C13—C24	119.5 (3)	C38—C39—C33	120.8 (2)
N3—C14—C15	122.1 (3)	C38—C39—C25	117.1 (2)
N3—C14—H14	118.9	C33—C39—C25	122.0 (2)
C15—C14—H14	118.9		
			
O2—S1—O1—Ni1	162.23 (13)	C13—N3—C14—C15	−0.3 (4)
O3—S1—O1—Ni1	33.92 (19)	Ni1—N3—C14—C15	−175.3 (2)
C27—S1—O1—Ni1	−78.20 (17)	N3—C14—C15—C16	−2.1 (5)
O9—Ni1—O1—S1	−16.47 (16)	C14—C15—C16—C17	1.8 (5)
N3—Ni1—O1—S1	−113.08 (16)	C15—C16—C17—C13	0.7 (4)
N1—Ni1—O1—S1	154.83 (16)	C15—C16—C17—C18	−178.7 (3)
N2—Ni1—O1—S1	75.11 (16)	N3—C13—C17—C16	−3.3 (4)
N4—Ni1—O1—S1	−91.4 (5)	C24—C13—C17—C16	176.5 (3)
O9—Ni1—N1—C2	163.0 (6)	N3—C13—C17—C18	176.2 (3)
N3—Ni1—N1—C2	−3.1 (3)	C24—C13—C17—C18	−4.0 (4)
N2—Ni1—N1—C2	−179.2 (3)	C16—C17—C18—C19	−177.4 (3)
N4—Ni1—N1—C2	−83.4 (3)	C13—C17—C18—C19	3.2 (4)
O1—Ni1—N1—C2	88.2 (3)	C17—C18—C19—C20	0.0 (5)
O9—Ni1—N1—C1	−15.1 (7)	C18—C19—C20—C24	−2.3 (5)
N3—Ni1—N1—C1	178.8 (2)	C18—C19—C20—C21	177.3 (3)
N2—Ni1—N1—C1	2.8 (2)	C24—C20—C21—C22	−0.4 (5)
N4—Ni1—N1—C1	98.5 (2)	C19—C20—C21—C22	179.9 (3)
O1—Ni1—N1—C1	−89.9 (2)	C20—C21—C22—C23	−0.5 (5)
O9—Ni1—N2—C11	−3.0 (3)	C24—N4—C23—C22	−1.7 (4)
N3—Ni1—N2—C11	153.5 (6)	Ni1—N4—C23—C22	−178.6 (2)
N1—Ni1—N2—C11	179.8 (3)	C21—C22—C23—N4	1.6 (5)
N4—Ni1—N2—C11	84.6 (3)	C23—N4—C24—C20	0.7 (4)
O1—Ni1—N2—C11	−93.2 (3)	Ni1—N4—C24—C20	178.1 (2)
O9—Ni1—N2—C12	174.7 (2)	C23—N4—C24—C13	179.3 (2)
N3—Ni1—N2—C12	−28.8 (7)	Ni1—N4—C24—C13	−3.2 (3)
N1—Ni1—N2—C12	−2.6 (2)	C21—C20—C24—N4	0.4 (4)
N4—Ni1—N2—C12	−97.7 (2)	C19—C20—C24—N4	180.0 (3)
O1—Ni1—N2—C12	84.4 (2)	C21—C20—C24—C13	−178.2 (3)
O9—Ni1—N3—C14	−99.1 (3)	C19—C20—C24—C13	1.4 (4)
N1—Ni1—N3—C14	78.7 (3)	N3—C13—C24—N4	2.9 (4)
N2—Ni1—N3—C14	104.6 (7)	C17—C13—C24—N4	−176.9 (2)
N4—Ni1—N3—C14	174.6 (3)	N3—C13—C24—C20	−178.4 (3)
O1—Ni1—N3—C14	−8.8 (2)	C17—C13—C24—C20	1.8 (4)
O9—Ni1—N3—C13	85.70 (19)	O7—C25—C26—C31	161.8 (3)
N1—Ni1—N3—C13	−96.47 (19)	C39—C25—C26—C31	−18.4 (4)
N2—Ni1—N3—C13	−70.6 (7)	O7—C25—C26—C27	−13.5 (5)
N4—Ni1—N3—C13	−0.56 (18)	C39—C25—C26—C27	166.2 (3)
O1—Ni1—N3—C13	176.04 (18)	C31—C26—C27—C28	−1.8 (4)
O9—Ni1—N4—C23	81.9 (3)	C25—C26—C27—C28	173.5 (3)
N3—Ni1—N4—C23	179.1 (3)	C31—C26—C27—S1	175.3 (2)
N1—Ni1—N4—C23	−89.8 (3)	C25—C26—C27—S1	−9.4 (4)
N2—Ni1—N4—C23	−9.4 (3)	O2—S1—C27—C28	−111.8 (3)
O1—Ni1—N4—C23	157.1 (4)	O3—S1—C27—C28	8.6 (3)
O9—Ni1—N4—C24	−95.10 (19)	O1—S1—C27—C28	125.4 (2)
N3—Ni1—N4—C24	2.06 (18)	O2—S1—C27—C26	71.0 (3)
N1—Ni1—N4—C24	93.17 (19)	O3—S1—C27—C26	−168.6 (3)
N2—Ni1—N4—C24	173.62 (18)	O1—S1—C27—C26	−51.8 (3)
O1—Ni1—N4—C24	−20.0 (6)	C26—C27—C28—C29	0.3 (5)
C2—N1—C1—C5	−0.8 (5)	S1—C27—C28—C29	−177.1 (3)
Ni1—N1—C1—C5	177.5 (2)	C27—C28—C29—C30	1.2 (6)
C2—N1—C1—C12	179.1 (3)	C28—C29—C30—C31	−1.1 (5)
Ni1—N1—C1—C12	−2.6 (3)	C29—C30—C31—C26	−0.4 (5)
C1—N1—C2—C3	1.3 (5)	C29—C30—C31—C32	−179.7 (3)
Ni1—N1—C2—C3	−176.7 (3)	C27—C26—C31—C30	1.9 (4)
N1—C2—C3—C4	−0.1 (6)	C25—C26—C31—C30	−173.7 (3)
C2—C3—C4—C5	−1.7 (6)	C27—C26—C31—C32	−178.9 (3)
C3—C4—C5—C1	2.1 (6)	C25—C26—C31—C32	5.5 (4)
C3—C4—C5—C6	−177.9 (4)	C30—C31—C32—O8	15.4 (5)
N1—C1—C5—C4	−0.9 (5)	C26—C31—C32—O8	−163.9 (3)
C12—C1—C5—C4	179.2 (3)	C30—C31—C32—C33	−166.1 (3)
N1—C1—C5—C6	179.1 (3)	C26—C31—C32—C33	14.7 (4)
C12—C1—C5—C6	−0.8 (5)	O8—C32—C33—C39	157.1 (3)
C4—C5—C6—C7	−178.5 (4)	C31—C32—C33—C39	−21.4 (4)
C1—C5—C6—C7	1.5 (5)	O8—C32—C33—C34	−19.8 (5)
C5—C6—C7—C8	−0.8 (5)	C31—C32—C33—C34	161.8 (3)
C6—C7—C8—C9	178.6 (3)	C39—C33—C34—C36	−3.0 (4)
C6—C7—C8—C12	−0.6 (5)	C32—C33—C34—C36	173.8 (3)
C12—C8—C9—C10	−2.4 (5)	C39—C33—C34—S2	168.3 (2)
C7—C8—C9—C10	178.3 (3)	C32—C33—C34—S2	−14.9 (4)
C8—C9—C10—C11	1.9 (5)	C36—C34—S2—O4	124.0 (3)
C12—N2—C11—C10	−1.5 (5)	C33—C34—S2—O4	−47.4 (3)
Ni1—N2—C11—C10	176.1 (2)	C36—C34—S2—O6	−112.2 (3)
C9—C10—C11—N2	0.1 (6)	C33—C34—S2—O6	76.3 (3)
C11—N2—C12—C8	0.9 (5)	C36—C34—S2—O5	4.5 (3)
Ni1—N2—C12—C8	−177.1 (2)	C33—C34—S2—O5	−166.9 (3)
C11—N2—C12—C1	180.0 (3)	C33—C34—C36—C37	0.6 (5)
Ni1—N2—C12—C1	2.0 (3)	S2—C34—C36—C37	−171.4 (3)
C9—C8—C12—N2	1.0 (5)	C34—C36—C37—C38	1.8 (5)
C7—C8—C12—N2	−179.7 (3)	C36—C37—C38—C39	−1.7 (5)
C9—C8—C12—C1	−178.0 (3)	C37—C38—C39—C33	−0.7 (5)
C7—C8—C12—C1	1.3 (4)	C37—C38—C39—C25	176.9 (3)
N1—C1—C12—N2	0.4 (4)	C34—C33—C39—C38	3.1 (4)
C5—C1—C12—N2	−179.7 (3)	C32—C33—C39—C38	−173.9 (3)
N1—C1—C12—C8	179.5 (3)	C34—C33—C39—C25	−174.4 (3)
C5—C1—C12—C8	−0.6 (5)	C32—C33—C39—C25	8.5 (4)
C14—N3—C13—C17	3.1 (4)	O7—C25—C39—C38	13.8 (4)
Ni1—N3—C13—C17	178.8 (2)	C26—C25—C39—C38	−166.0 (3)
C14—N3—C13—C24	−176.8 (2)	O7—C25—C39—C33	−168.6 (3)
Ni1—N3—C13—C24	−1.0 (3)	C26—C25—C39—C33	11.6 (4)

### Hydrogen-bond geometry (Å, °)

**Table:**

*D*—H···*A*	*D*—H	H···*A*	*D*···*A*	*D*—H···*A*
O9—H9A···O3	0.85	1.90	2.704 (3)	156
O9—H9B···O5^i^	0.85	1.97	2.823 (3)	179

Symmetry codes: (i) *x*, *y*−1, *z*.
